# Hypermobility of joints in dancers

**DOI:** 10.1371/journal.pone.0212188

**Published:** 2019-02-22

**Authors:** Marlena Skwiot, Grzegorz Śliwiński, Steve Milanese, Zbigniew Śliwiński

**Affiliations:** 1 Faculty of Medicine and Health Sciences, Jan Kochanowski University in Kielce, Kielce, Poland; 2 Faculty of Biomedical Engineering, Technische Universität Dresden, Dresden, Germany; 3 School of Health Science, University of South Australia, Adelaide, Australia; Shenandoah University College of Arts and Sciences, UNITED STATES

## Abstract

**Objectives:**

The current understanding of hypermobility and its diagnostic criteria is still insufficient to create a complete and systematic clinical presentation of the disorder. The objective of this study was to assess the prevalence of joint hypermobility syndrome (JHS) amongst a cohort of jazz dancers, by analyzing its presence in accordance with a number of diagnostic criteria, and to verify potential risk factors for joint hypermobility in jazz dancers.

**Methods:**

77 jazz dancers from the Polish Dance Theater were examined (58 female and 19 male). The prevalence of JHS was assessed using the following diagnostic tools: a structured interview, Beighton score, Grahame & Hakim questionnaire, and Sachse’s criteria, in the modified version proposed by Kapandji.

**Results:**

The prevalence of JHS in this cohort of jazz dancers differed significantly, depending on which criteria were adopted (p = 0.001) with Beighton score, Grahame & Hakim questionnaire, and Sachse’s criteria identifying 64.9%, 74% and 59.7% of the sample as JHS respectively. Hypermobility was significantly more prevalent in women than men (p < 0.05).

**Conclusions:**

This study demonstrated a significant prevalence of joint hypermobility in jazz dancers and corroborates the findings of other researchers, indicating the need for unified diagnostic criteria for JHS in dancers.

## Introduction

Hypermobility is a term that describes where a joint is able to move beyond its normal range of movement (ROM), usually secondary to laxity of the passive ligamentous/capsular constraints [1]. This condition should not be confused with flexibility which is a function of both joint ROM and muscle length. Joint Hypermobility Syndrome (JHS) refers to a condition where four or more joints are able to move beyond their normal physiological range. Steinberg et al. (2016) reported that the JHS prevalence was significantly higher among dancers compared with non-dancer control subjects [2].

Due to dance technical requirements, stretching has become one of the main training routines of many professional dancers across the spectrum of dance types [3]. Whilst the aim of stretching in dancers is to increase joint range of motion beyond physiological norms, thus allowing the dancers to achieve their desired dance aesthetics [4], there remains some debate in the literature about the efficacy of this training. Steinberg et al. (2012) [5] in a cross sectional study of 1320 female dancers (involved in classical ballet, modern dance, jazz, or composition) and 226 nondancers, aged 8 to 16 years identified that the difference in ROM between dancers and nondancers varied with specific joints, type of movement, and age. For a number of measures such as ankle and foot plantar flexion (pointe), ankle plantar flexion, and hip external rotation ROM dancers maintained or increased their ROM, compared to nondancers over this age range.

Researchers, who devoted their academic careers to the problem of excessive flexibility in dancers, have always faced a puzzling question: is hypermobility genetically determined or is it simply the result of the strenuous training routines that dancers undergo since childhood? Unfortunately, this question still remains unanswered [4]. Furthermore, there are conflicting opinions in the literature on whether excessive flexibility in dancers is a benefit or a problem. Some researchers have reported evidence of a correlation between joint hypermobility and increased risk of injury, identifying JHS as the underlying cause of impaired coordination and poor physical performance, chronic fatigue, and mental strain [6, 7]. Other authors have suggested that the JHS often found in dancers is an asset more than a liability [8] with excessive joint laxity in children often perceived by dance schools as a marker of future success in professional dancing.

However, the struggles with training intensity and peer pressure that some young dancers have to face frequently prove to be too arduous for them to handle. A prolonged adaptation period and low body awareness during their initial training may lead to musculoskeletal damage [9].

The Beighton 9 point system is frequently used for clinical diagnosis of JHS [10]. It can also be supplemented with a 5-part questionnaire developed by Hakim and Grahame, which enables a swift clinical diagnosis [11]. In Poland, the 13 tests of joint motion developed in accordance with the methodology presented by Sachse, and modified by Kapandji are also used for diagnosing joint hypermobility in dancers. The criteria include an evaluation of the motion of peripheral joints and the spine column with regard to three categories [12–14]. Many researchers claim that traditional methods to diagnose joint hypermobility in dancers do not produce reliable results. Bird (2016) [8] reported that the current evidence base on JHS in dancers was flawed by methodological features in identifying joint hyper mobility which overlooked the special nature of dance. They suggested that the assessment of dancer hypermobility should include a broader number of joints and consider variables such as dance style. To date, no specialised criteria that would allow for accurate diagnosis of JHS in professional dancers have yet been developed. The current understanding of JHS and its diagnosis is still insufficient to create a complete clinical presentation of the disorder, and therefore its symptoms are often ignored [10]. Lack of a unified criteria for evaluating joint hypermobility is a considerable obstacle to establishing proper diagnosis, especially in dancers.

The method in which the tests are administered, also plays an important role in the process of diagnosing JHS in dancers. For instance, an increased range of joint motion is observed when the study subjects participate in a warm-up session before the measurement proper. Furthermore, the tests will yield different measurements if they are administered after the training session, due to muscle fatigue [9].

The objective of this study was to assess the prevalence of JHS in a cohort of jazz dancers using a number of different commonly used diagnostic criteria. An equal aim was to check potential risk factors for hip joint hypomobility in jazz dancers. The null hypothesis assumes a uniform distribution of the analyzed variables among the study groups (patients with JHS and healthy individuals).

## Materials and methods

### Ethics statement

The study was conducted between 2013 and 2014, on the basis of a consent issued by the Bioethics Commission of the Faculty of Health Sciences, at The Jan Kochanowski University in Kielce, as of 11.03.2013.

Participation in the study was voluntary and its procedure was designed in accordance with the provisions of the Act of 29 August 1997 on the Protection of Personal Data (Dz.U. Nr 133 poz. 883). Verbal consent was obtained to conduct the research from the participants. The director of the Kielce Dance Theater has agreed in writing to conduct the study. The authors confirm that all ongoing and related trials for this drug/intervention are registered. The name of the trial’s registry and the registration number: ISRCTN 31838963. This study was not registered before the registration of participants due to the absence of such requirements in the country where the study was conducted.

A copy of the report from the trial protocol was submitted as an auxiliary information file (S1 and S2 Files). The authors presented the results regarding the diagnosis of hypermobility syndrome. Part of the research on the use of kinesiology taping has been abandoned.

### Participants

**Fig 1:**
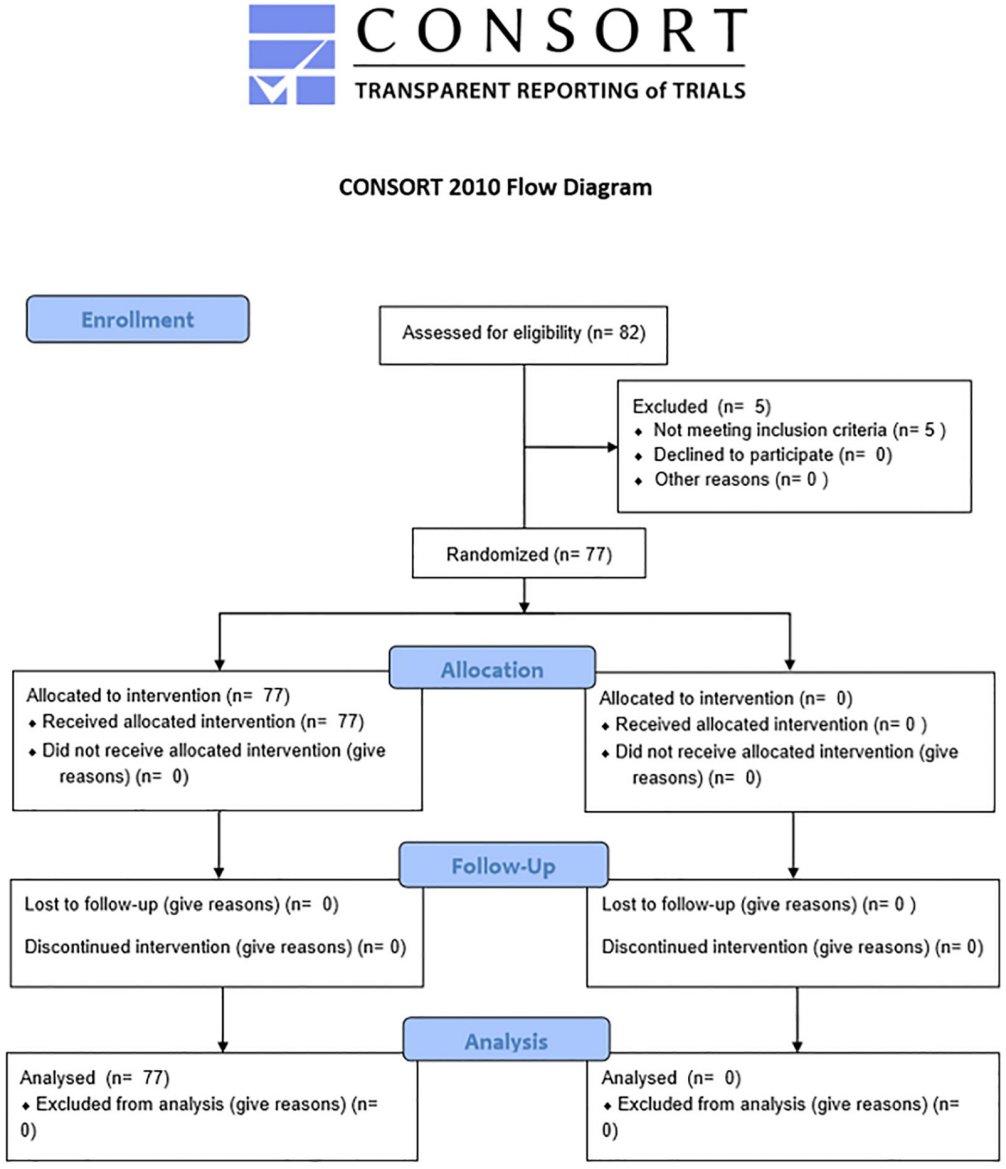
The CONSORT flow diagram.

77 jazz dancers from the Polish Dance Theater were recruited (58 female and 19 male). Research inclusion criteria were as follows: a) age between 18 and 25 years old b) at least 3 years of dancing experience, c) jazz dance as the dominant dance style. The study was conducted in the middle of the dance season. Fig 1 shows the CONSORT flow diagram.

### Methods of evaluating symptoms

Data on the participants, including dance history and current dance performance, and the JHS symptoms they experienced was collected through a structured interview. Specific questions were asked about injuries, symptoms typical for hypermobility syndrome such as chronic spinal pain, temporomandibular joint disorders. In addition, participants were asked about out-of-joint symptoms such as possible skin lesions, myopia, varicose veins. Interviews were carried out by the same physiotherapist.

### Methods of evaluating hypermobility

All subjects underwent anthropometric assessment with measures taken of standing height, using a stadiometer, and weight, using a standard set of scales. The presence of joint hypermobility syndrome was evaluated using the following diagnostic tools: Beighton score [10], Grahame & Hakim questionnaire [11], and Sachse’s criteria [12], in the version modified by Kapandji.

ROM, which were involved in the Beightons score and the Sasche’s scale (described in detail below), was measured using a Baseline digital inclinometer. The Baseline inclinometer has been shown to be more reliable than the standard universal goniometer [15]. All measures were taken by a licensed physiotherapist with a master’s degree, who used to be an active dancer in the study group.

#### Beighton score

The Beighton score [10] evaluates the prevalence of the following symptoms:

1. Passive dorsiflexion of the little fingers beyond 90°;
2. Passive apposition of the thumbs to the flexor aspects of the forearm;
3. Hyperextension of the elbows beyond 10°;
4. Hyperextension of the knee beyond 10°;
5. Forward flexion of the trunk with knees fully extended so that the palms of the hands rest flat on the floor.

A positive result in each of the tests adds 1 point to the overall score. A minimum of 4 out of 9 points is considered to be an indicator of JHS. Whilst there is dispute in the literature about the optimal cut-off score, with some authors considering a Beightons score of >5 to be indicative of JHS, Beighton’s original work recommended a cut off score of 4 (Beighton). Der Giessen et al [14] recommended that up to the age of 9 a score of 5 out of 9 be used to identify JHS but after 10 years of age the cut off score should be 4 out of 9. Boyle et al. [16] reported the percentage agreement and the Spearman rho for intrarater and interrater reliability of Beighton composite scores (1–9) was 69% and .86 and 51% and .87, respectively.

#### Hakim & Grahame questionnaire

Hakim and Grahame questionnaire [11] consists of five questions about the currently experienced symptoms and their prevalence in the past:

1. Can you now (or could ever) place your hands flat on the floor without bending your knees?
2. Can you now (or could ever) bend your thumb to touch your forearm?
3. As a child did you amuse your friends by contorting your body into strange shapes or could you do the splits?
4. As a child or teenager did your shoulder or kneecap dislocate on more than one occasion?
5. Do you consider yourself double-jointed?

A minimum of 2 positive answers is considered to be an indicator of JHS, with diagnostic sensitivity at 80–85% [11].

#### Sachse’s scale

The 13 tests developed by Sachse [12] use three levels of mobility evaluation:

A—Joint mobility is within the range from hypomobile to normal;
B—Normal or slightly hypermobile joints;
C—Marked joint mobility, hypermobility.

A positive score in at least 7 out of 13 tests allows for a positive diagnosis of JHS. The tests use the following methods to measure and classify joint mobility:

1. Lumbar spine retroflexion–starting position: lying prone, elbows bent, hands placed flat on the floor at the height of the shoulders, forearms placed along the long axis of the body. Motion: lifting the upper body by the pressure of the arms, raising it as far as the lumbar spine. Extension degree of the elbow joint: A. <90°; B. 90°-120°; C. >120°.
2. Lumbar spine anteflexion–starting position: Standing upright. Motion: bending forward to touch the floor with the hands, knee joints extended. The patient: A. touches the floor with the tips of her fingers; B. touches the floor with her knuckles (i.e. the metacarpophalangeal joints are bent) C. entire hands are placed on the floor; the chest is placed against the thighs.
3. Lumbar side-bending–starting position: standing upright. Motion: side-bending. The plumb line from the fold of the contralateral axilla: A. reaches no further than the intergluteal cleft; B. reaches the middle of the buttock on the opposite side; C. reaches beyond the lateral aspect of the buttock.
4. Trunk rotation–starting position: sitting astride a chair without back support, hands placed on the nape of the neck, elbows together. Motion: turning left and right in succession: A. <50°; B. 50°-70°; C. >70°.
5. Rotation of the head and neck–starting position: sitting on a chair, cervical spine retracted. Rotation in one direction: A. <70°; B. 70°-90°; C. >90°.
6. Passive dorsal hyperextension of metacarpophalangeal joints—assessment of average angle between the long axis of the forearm and the little finger: A. <45°; B. 45°-60°; C. >60°.
7. Passive apposition of thumb-to-flexor aspect of forearm—A. the thumb does not touch the forearm; B. the thumb touches the forearm slightly; C. the thumb easily touches the forearm, can be placed against it.
8. Elbow joint extension–starting position: sitting upright, shoulder and elbow joints bent at an internal angle of 90°, forearms aligned and touching, hands with palms facing upward. Motion: extending elbow joints without separating the forearms: A. >80°; B. 80°-65°; C. <65°.
9. Shoulder mobility within the shoulder girdle–starting position: sitting upright on a chair. Motion: bringing the elbow joint towards the contralateral shoulder: A. the elbow reaches the midline of the body; B. the elbow crosses the midline of the body and moves towards the contralateral shoulder; C. the elbow touches the contralateral shoulder.
10. Shoulder mobility–starting position: standing upright. Motion: attempting to make both hands meet diagonally behind the back: A. the fingers do not touch; B. the fingers come into contact or overlap at the height of the first phalanx bone; C. Entire hands overlap.
11. Passive extension of knee joint–lying supine: A. full extension; B. Hyperextension <10°; C. Hyperextension >10°.
12. Passive abduction of hip joint–lying supine: A. <40°; B. 40°-50°; C. >50°.
13. Rotation of hip joint–combination of passive internal and external rotation in one joint, lying supine A. <90°; B. 90°-120°; C. >120°.

### Statistical methods

To verify the alternative hypothesis (H1), the results of the study were analyzed using statistical tools Microsoft Excel 2013 was used for statistical analysis. The variables were analyzed with descriptive statistics (mean, standard deviation). A chi-square test of independence was used to determine if there was a significant relationship between nominal (categorical) variables. As the data was not presumed to be normally distributed, the Mann-Whitney U test was used to compare male and female participants, and to compare participants with JHS and without JHS, in accordance with the analyzed variables. A significance level of P<0.05 was set for all statistical analyses.

## Results

The descriptive anthropometric descriptive statistics were as follows: age 18–25 years (mean = 20.03 years, SD = 2.67), height (mean = 1.70 m, SD = 0.06), weight (mean = 57.95 kg, SD = 10.02), dancing experience (mean = 9.47 years, SD = 4.52), frequency of practice sessions (mean = 13.51 h/week, SD = 15.04).

**Fig 2:**
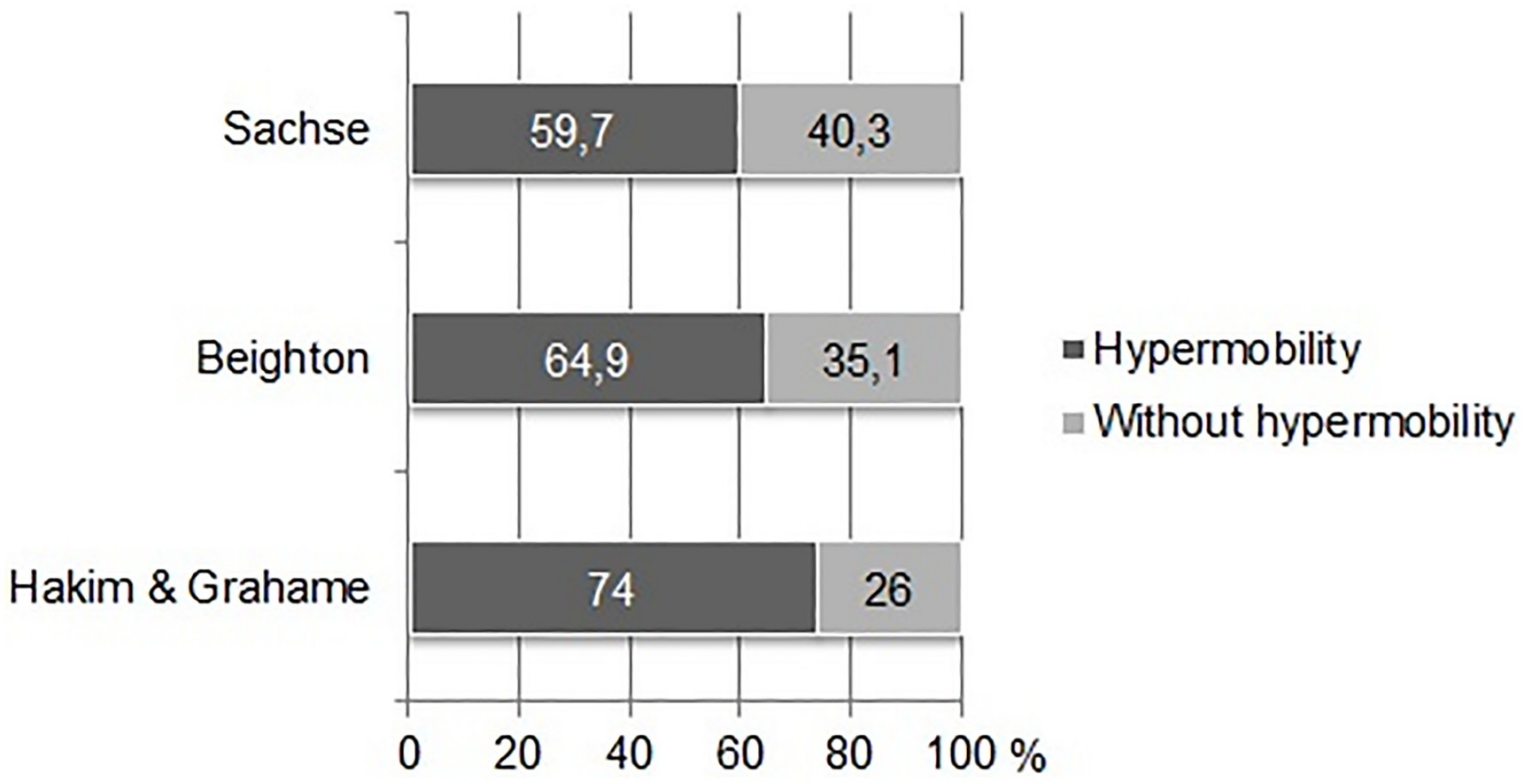
Percentage frequency distribution of JHS prevalence, according to Sachse, Beighton, and Hakim & Grahame.

Fig 2 shows the percentage frequency distribution of JHS, as evaluated using the Beighton score, Grahame & Hakim questionnaire, and Sachse’s criteria, in the version modified by Kapandji.

The prevalence of JHS in the analyzed sample differed significantly, depending on the criteria of the analysis *χ*^*2*^(2) = 13.786; p = 0.001. According to the Sachse’s criteria, in the version modified by Kapandji, 59.7% of participants were diagnosed with JHS. On the other hand, Beighton’s score yielded a result of 64.9%, and Hakim & Grahame questionnaire a result of 74%.

**Fig 3:**
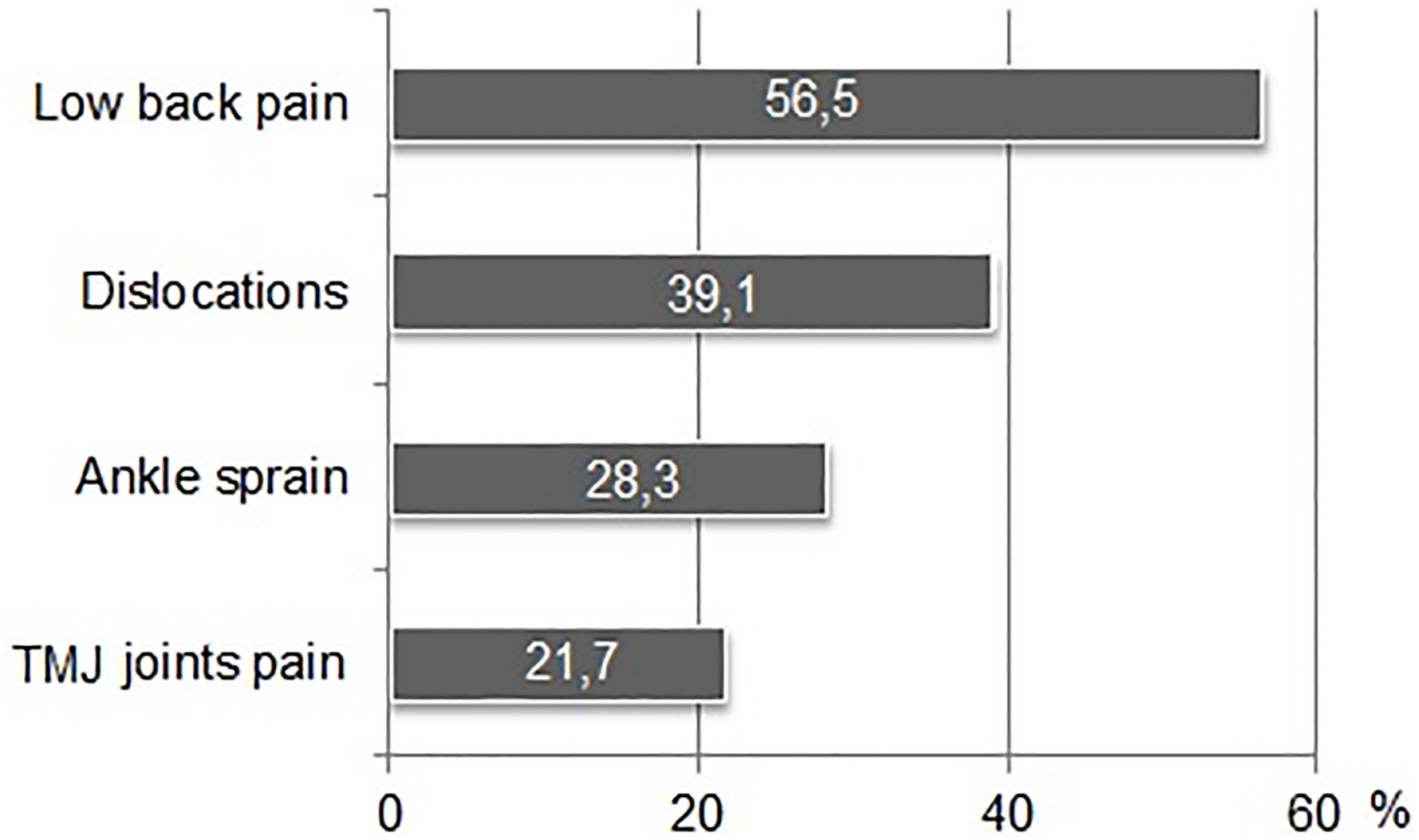
Percentage frequency distribution of the prevalence of JHS symptoms.

On the basis of the structured interview, 46 participants, who were diagnosed with hypermobility using Sachse’s scale, revealed symptoms which may be related to JHS (Fig 3), with chronic pain in the lumbar spine being the dominant one (56.5%) (60%—Beighton; 63, 1%—Hakim & Graham). Reporting sensations of dislocations of the lower limbs was the second most frequently occurring symptom (39.1%). 28.3% of the participants reported having suffered an ankle sprain in the past. Temporomandibular joint (TMJ) pain was the least frequently experienced symptom among the participants.

Subjects with or without joint hypermobility differed in the incidence of joint symptoms typical of JHS, such as chronic low back pain χ2 (1) = 4.37; p = 0.036. No differences were found in other symptoms: temporal and temporal disorders χ2 (1) = 2.12; p = 0.145, twisting of the cube χ2 (1) = 1.72; p = 0.19, spine fracture χ2 (1) = 0.55; p = 0.458 and limb instability χ2 (1) = 2.8; p = 0.094. Non-arthritis symptoms in both groups were rare. There were no significant differences between the groups for myopia (χ2) = 0.12; p = 0.731 and skin lesions such as stretch marks, scars, dilution χ2 (1) = 0.55; p = 0.458.

**Fig 4:**
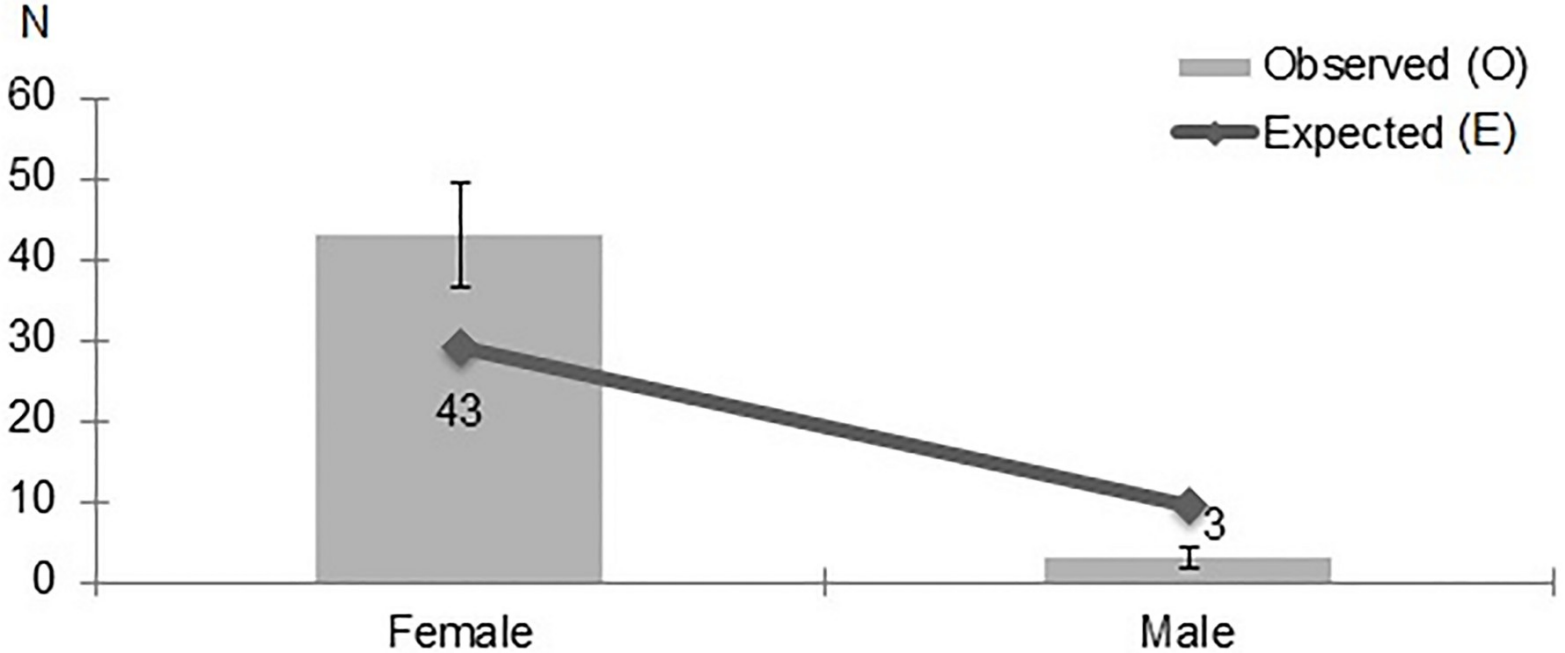
Gender distribution of JHS patients, as diagnosed with Sache’s scale.

**Fig 5:**
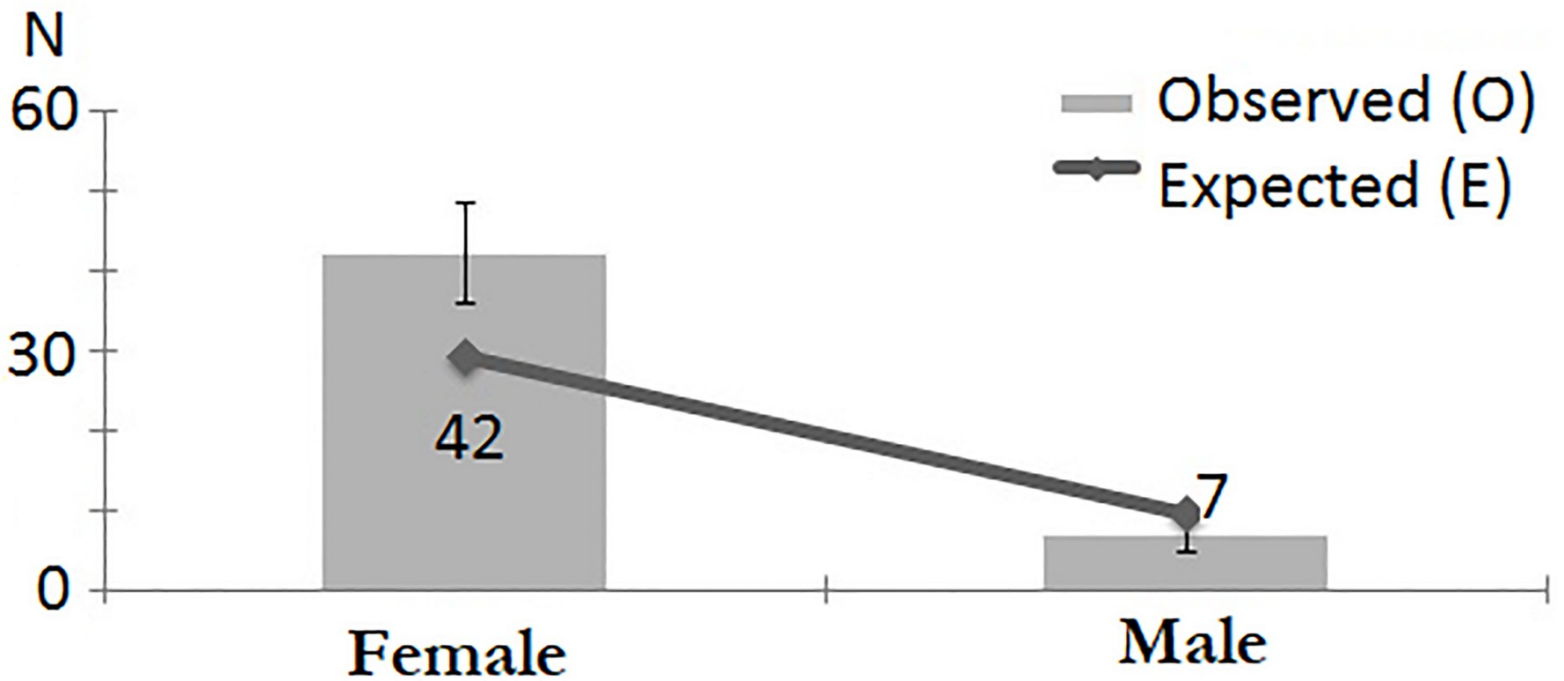
Gender distribution of JHS patients, as diagnosed with Beighton score.

**Fig 6:**
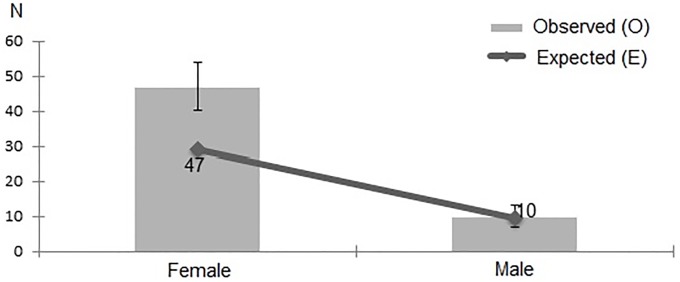
Gender distribution of JHS patients, as diagnosed with Hakim & Grahame criteria.

The chi-square test of independence showed a correlation between hypermobility and the participants’ gender. Hypermobility was significantly more prevalent in women than men, regardless of which diagnostic criteria were adopted: Sachse–*χ*^*2*^(1) = 11.206; p = 0.001 (Fig 4), Beighton–*χ*^*2*^(1) = 6.485; p = 0.011 (Fig 5), Hakim & Grahame–*χ*^*2*^(1) = 11.199; p = 0.001 (Fig 6).

Due to the differences in JHS prevalence in men and women, the male and female participants were compared with regard to the following variables: body mass [kg], height [m], dancing experience [years], frequency of practice sessions [h/week], using the Mann-Whitney U test. The results showed that, on average, women were thinner (M = 53.64, SD = 5.61 vs. M = 72.68, SD = 5.04; U = 3.5, p < 0.0001) and shorter (M = 166.96, SD = 3.61 vs. M = 178.68, SD = 4.27; U = 24.5, p < 0.0001) than men. No significant differences between men and women were found with regard to their dancing experience (M = 9.42, SD = 4.83 vs. M = 10.21, SD = 4.32; U = 403.5, p = 0.339) or frequency of practice sessions (M = 12.36, SD = 14.22 vs. M = 19.16, SD = 16.26; U = 355.5, p = 0.112).

The results of the initial analysis were used to compare patients diagnosed with JHS and healthy individuals, with regard to the following variables: BMI, dancing experience [years] and frequency of practice sessions [h/week]. The follow-up analysis used the Mann-Whitney U test (Table 1).

**Table 1:** Summary of the Mann Whitney U test results, as a comparison of selected variables between JHS patients and healthy individuals, in accordance with different evaluation criteria (n = 77).

JHS evaluation criterion	Variable	JHS N = 46		No JHS N = 31		Test statistics
		Mean	SD	Mean	SD	U
Sachse	BMI (Kg/m^2^)	19.31	1.83	21.09	2.30	401.5*
	Dancing experience (years)	9.78	4.78	9.00	4.13	673.5
	Frequency of practice sessions (per week)	13.04	14.37	14.19	16.21	611.0
Beighton	BMI (kg/m^2^)	19.69	2.17	20.54	2.37	525.0
	Dancing experience (years)	9.14	4.14	10.07	5.18	612.5
	Frequency of practice sessions (per week)	13.06	14.88	14.33	15.59	640.0
Hakim & Grahame	BMI (kg/m^2^)	19.66	2.01	21.07	2.45	377.0
	Dancing experience (years)	9.37	4.61	9.75	4.34	490.0
	Frequency of practice sessions (per week)	11.88	13.95	18.15	17.34	451.0

*Significant (p < 0.05)

The groups (patients diagnosed with JHS on the basis of Sachse’s test and the healthy individuals) differed significantly with regard to the BMI variable (U = 401.5, p = 0.002). No significant differences between the groups were found with regard to dancing experience and frequency of practice sessions (p > 0.05). The analysis showed no significant differences between groups, with regard to the Beighton score and Hakim & Grahame’s diagnostic criteria (p > 0.05).

## Discussion

Professional dancing often requires unique movement patterns that go beyond anatomical planes and standard biomechanical properties of the body, frequently causing strain to the musculoskeletal system. Ligaments laxity and joint capsules may lead to disturbed joint stability and put dancers at risk of injury [4, 17]. The purpose of this study was to assess the prevalence of joint hypermobility syndrome (JHS) amongst a cohort of jazz dancers, by analyzing its presence in accordance with a number of diagnostic criteria, and to verify potential risk factors for joint hypermobility in jazz dancers. Therefore, the null hypothesis, which assumes a uniform distribution of the analyzed variables among the study groups (patients with JHS and healthy individuals), should be rejected. This confirms the findings of other studies, which show female gender to be a risk factor in JHS [18]. Lower BMI was also found to be correlated with JHS, which goes against the reports of researchers who link joint hypermobility with a tendency for adult obesity. However, this link was established with regard to patients who did not practice sports [13].

This study corroborates the previous findings that show joint hypermobility syndrome to be diagnosed more frequently in dancers [19] than in the general population from 12% to 24% more prevalent, according to Stodolna [13]. The negative correlation between prevalence of JHS and the specificity of the adopted diagnostic criteria suggests that differences in the sporting pursuits have to be taken into account when evaluating JHS in athletes, and that appropriate diagnostic criteria for dance need to be selected.

The Sachse’s scale, in the version modified by Kapandji, presents itself as a useful diagnostic tool for hypermobility in dancers. It includes, apart from the standard battery of tests, mobility tests of the shoulder girdle, hip joints, and the spine column in different planes. According to Day et al. [18], 44% of dancers suffer from JHS (as diagnosed with the Beighton score), which is significantly less than was found in this study (64.9%).

Dancing often involves positions, both static and dynamic, that require maximal extension in the lumbar spine. When attempting to overcome individual limitations and achieve the desired dancing aesthetic, dancers, and especially beginners, frequently compensate deficiencies in their technique by increasing the angle of hip anteflexion and lumbar lordosis, which can be a cause of pain [20–22]. The fact that 56.5% of participants reported chronic pain (> 3 months) in the lumbar spine (Fig 2), is a clear indication that pain prevention measures for dancers suffering from joint hypermobility are probably needed. The JHS group was significantly different from the non-JHS group in terms of lumbar pain (56.5% vs. 25.8%). This claim also finds support in the literature [7, 23]. Briggs et al. [24] found no evidence of differences between dancers diagnosed with JHS and healthy dancers, with regard to back pain, disturbed lower limb stability, and ankle sprain.

Dysfunctions of the temporomandibular joint, which are commonly identified in JHS patients, proved to be least relevant to this study [25] reflecting the finding of Berger et al. [26] who found no significant correlation between TMJ dysfunction and JHS.

Expanding the understanding of JHS could help in planning the functional improvement process and adapting it to the specific causes of musculoskeletal dysfunctions among dancers. It can also improve their quality of life by alleviating the pain caused by excessive training regimes. Knowledge of the causes of problems, related to jhs, would allow to plan effective preventive actions and to apply a multi-faceted, comprehensive rehabilitation program.

The research had some limitations. The study was attended by all dancers from the dance theater, who met the inclusion criteria, which is why the number of the study group was limited. In addition, the study is limited only to a specific group of jazz dancers. There is a need to expand research to assess the importance of JHS criteria among dancers in other dance styles, as well as in other sports. Moreover, future avenues for research that deserves more attention includes confirmation of potential risk factors for joint hypermobility in dancers. There is also a low degree of control over the influence of external factors in research on complex human behavior.

## Conclusions

This study demonstrates a significant prevalence of joint hypermobility in jazz dancers. The significant differences in the results yielded by the different hypermobility tests confirm the need for a unified diagnostic criteria for JHS. Researchers should consider creating separate diagnostic criteria for participants who practice specialsied activities such as dance. This study has identified female gender and low BMI as risk factors for joint hypermobility in jazz dancers.

## Supporting information

S1 Table TREND Checklist. (PDF)

S1 File A copy of the trial study protocol (in Polish). (DOCX)

S2 File A translation of the trial study protocol. (DOCX)
