# Supplementary material for: Hypermobility of joints in dancers
Source: PLoS One. 2019 Feb 22;14(2):e0212188. doi: 10.1371/journal.pone.0212188 (PMC6386248; doi:10.1371/journal.pone.0212188)
Supplement: S1 File — (DOCX) [file pone.0212188.s002.docx]

Marlena Drężewska Załącznik nr1

ul. Piastowska 20B (Wypełnia Komisja Bioetyczna)

07-407 Czerwin Data wniosku_________________

Nr akt_______________________

### WNIOSEK

Do Komisji Bioetycznej w sprawie wyrażenia opinii na przeprowadzenie
oceny badania naukowego.

1. **Badaniem kieruje dr hab. n. med. prof. nadz. Zbigniew Śliwiński**.

Eksperyment zostanie przeprowadzony w Kieleckim Teatrze Tańca, Plac Moniuszki 2B
25-334 Kielce.

Miejsce realizowania pracy jest podyktowane głównie możliwością pozyskania grupy badanej do przeprowadzenia eksperymentu badawczego. Wnioskodawca samodzielnie wykona badanie.

**2. Kierownik tematu (wnioskodawca) i członkowie zespołu badawczego:**

Wnioskodawca: Marlena Drężewska - mgr fizjoterapii

Zespół:

Prof. Zbigniew Śliwiński

Dr Wojciech Kiebzak

Dr Małgorzata Starczyńska

Dr Marek Woszczak

Dr Marek Kiljański

**3. Tytuł projektu:**

Wpływ aplikacji metody dynamicznego plastrowania na dysfunkcje narządu ruchu u tancerzy.

**4. Proponowany termin zakończenia badań.**

2013r.

**5. Dynamiczne plastrowanie / Kinesiology Taping**

Twórcą oraz propa­gatorem rewolucyjnej metody leczenia nazwa­nej Kinesiology Tapingiem jest dr Kenzo Kase - japoński chiropraktyk, Prezydent Towarzystwa Kinesio Tapingu oraz Narodowej Szkoły Chiropraktyków w Japonii, absolwent Uniwersytetu w Meiji oraz Narodowej Szkoły Chiropraktyków w Chicago.

Założeniem metody było, aby zastosowana terapia mogła od­działywać na pacjenta nie tylko w czasie wizyty, ale i po jej zakoń­czeniu, albowiem pozytywne działa­nia fizjoterapeutyczne zanikają często wraz z upływem czasu.

Idea stworzenia akceptowalnego przez organizm wspomagania i tera­pii wymagała odpowied­nich materiałów. Początkowe próby wykorzystania taśm nierozciągliwych u sportowców i chorych nie dawały pożądanych rezultatów. Został opracowany, w toku wieloletnich doświadczeń, plaster o naz­wie Kinesiology Tape, którego grubość, ciężar właściwy i rozciągliwość - w zakresie 130-140%, są zbliżone do parametrów ludzkiej skóry. Jest on wodoodporny, przepuszczalny dla powietrza, co pozwala na niezakłó­coną wymianę cieplną. Podstawa działania kinesiotapin­gu jest zupełnie odmienna od dzia­łania tapingu sportowego.

Kinesiology Taping to przede wszys­tkim oddziaływanie sensoryczne. Aplikacje pozwalają na zachowanie pełnego zakresu ruchu, powodują normalizację napięcia mięśniowego, aktywowanie uszkodzonych mięśni, zmniejszenie bólu, a także likwidują zastoje i obrzęki limfatyczne, kory­gują ułożenia powięzi i skóry oraz popra­wiają mikrokrążenie.

Każda aplikacja uwarunkowana jest wskazaniami klinicznymi. Plaster Kinesiology Tape przyklejony we właściwy sposób najczęściej w pozycji, w której skóra i powięź jest napięta tworzy pofałdowania powierzchni, zwiększając przestrzeń pomiędzy skórą właściwą a powięzią, co usprawnia mikrokrążenia krwi i limfy oraz aktywuje pro­ces samoleczenia. Współistniejące przy wielu scho­rzeniach zaburzenie ruchomości powięzi jest mechanizmem utrudnia­jącym samoleczenie. Dlatego od­działywanie na zmniejszenie nacisku ułatwi przepływ podskórny i wza­jemną przesuwalność tkanek.

W metodzie Kinesiology Taping wykorzystywane są następujące techniki: mięśniowe, więzadłowe, powięziowe, limfatyczne, funkcjonalne oraz korekcyjne.

Kinesiology Taping wpływa na funkcje mięśni poprawiając ich funkcjonowanie szczególnie wtedy, gdy są nadmiernie rozciągnięte, zmniejszając ból i wzmożone napię­cie, redukując zmęczenie oraz zwiększa­jąc zakres ruchu w stawach objętych ich działaniem.

Dzięki tej metodzie fizjote­rapia zyskała nowe skuteczne narzędzie terapeutyczne. Obserwując możliwości, jakie niesie metoda, znajdujemy zastosowanie dla obsza­rów dotychczas niedostępnych dla fizjoterapii.

**Zespół hipermobilności konstytucjonalnej.**

Zespół hipermobilności konstytucjonalnej (ZHK) (ang. Hypermobility Syndrome) to uogólniona, wrodzona niewydolność tkanki łącznej całego organizmu, związana z zaburzeniami proporcji występowania kolagenu typu I i III, powodując objawy w narządzie ruchu oraz szereg nieprawidłowości współtowarzyszących z innych układów zawierających tkankę łączną.

Głównymi objawami klinicznymi w obrębie narządu ruchu (hipermobilność konstytucjonalna stawów) są wiotkość więzadeł oraz torebek stawowych stawów obwodowych i międzywyrostkowych kręgosłupa oraz zwiększony, w odniesieniu do normy, zakres ruchów stawów, powodując obniżenie wydolności mechanicznej i stabilizacyjnej krążków międzykręgowych i stawów.

W diagnostyce klinicznej hipermobilności konstytucjonalnej stosuje się różne skale, m. in. Sachse’go, Beinghton’a, Hakim’a i Graham’a.

Zwiększona ruchomość w stawach może być zmianą wrodzoną, ale także nabytą – w przebiegu wieloletniego intensywnego treningu, w wyniku przebytego urazu, a także innych chorób tkanki łącznej i/lub układu ruchu. Największy problem stanowi diagnostyka zespołu hipermobilności konstyutucjonalnej u osób, u których zwiększony zakres ruchów w stawach stanowi efekt wieloletniego treningu np. u tancerzy czy gimnastyków.

**Hipotezy badawcze:**

W związku z częstym występowaniem hipermobliności konstytucjonalnej bądź nabytej u tancerzy oraz różnego rodzaju dysfunkcji narządu ruchu połączonych występowaniem bólu - często uniemożliwiającym uczestnictwo tancerzy w treningu czy spektaklach postanowiono zweryfikować następujące hipotezy badawcze:

1. U większości tancerzy w różnych grupach wiekowych obserwuje się występowanie hipermobilności konstytucjonalnej bądź nabytej.
2. Dostosowanie odpowiednich kryteriów oceny hipermobilności stawowej jest niezbędne do tworzenia indywidualnego programu usprawniania u tancerzy.
3. Aplikacje dynamicznego plastrowania powodują normalizację napięcia mięśniowego u tancerzy.
4. Dynamiczne plastrowanie zmniejsza poziom bólu odczuwanego przez tancerzy.
5. Techniki metody Kinesiology Taping poprawiają stabilność nadruchomych stawów obwodowych u tancerzy.
6. Dysfunkcje narządu ruchu wpływają na zmianę poziomu nastroju u tancerzy.

**Materiał badań:**

Badaniu zostanie poddana grupa ok. 80 tancerzy obu płci. Badani będą tancerzami Kieleckiego Teatru Tańca.

**Metoda badań:**

Badanie będzie obejmowało:

1. Wywiad personalny.
2. Ocenę postawy ciała tancerzy ze szczególnym uwzględnieniem statyki miednicy.
3. Wykonanie 13 testów wg Sachsego określających bądź nie hipermobilność konstytucjonalną, z uwzględnieniem trzech kategorii oceny:

- kategoria A – ruchomość stawów mieści się w zakresie od hipomobilności do ruchomości prawidłowej.
- kategoria B – ruchomość normalna lub niewielka hipermobliność.
- kategoria C – ruchomość znacznie zwiększona, hipermobilność.

1. Wykonanie testów w skali Beighton’a.
2. Wykonanie testów Hakima i Grahama.
3. Wykonanie testów screeningowych metody dynamicznego plastrowania dla obszaru dolnej części ciała:
   - test Linder 2,
   - test uciskowy brzucha
   - test Patric’a Fabre
   - test SLR.
4. Pomiar siły mięśniowej przy użyciu aparatu MICROFET 2 – Hoggan Health Industries, Inc.

Podczas testu badany znajduje się w pozycji izolowanej. Badający jedną ręką stabilizuje pozycję pacjenta, a drugą ręką trzyma przetwornik aparatu na badanym mięśniu. Badany wykonuje napięcie wskazanego mięśnia oraz typowy ruch dla tego mięśnia.

Badanie będzie dotyczyć następujących mięśni:

- m. najszerszy grzbietu

- m. czworoboczny lędźwi

- m. gruszkowaty

- m. pośladkowy średni

- m. pośladkowy wielki

- m. biodrowo - lędźwiowy

- m. prosty uda

- m. dwugłowy uda

- m. półbłoniasty

- m. półścięgnisty

- m. naprężacz powięzi szerokiej

- m. przywodziciel krótki

- m. przywodziciel długi

- m przywodziciel wielki.

1. Pomiar zakresu ruchów kończyny dolnej oraz części lędźwiowej kręgosłupa przy użyciu inklinometru cyfrowego. Zakres ruchów będzie określany metodą ISOM (International Standard Ortopedic Measurement), a wyniki będą zapisywane za pomocą systemu SFTR (Sagital, Frontal, Transverse, Rotation).
2. Ocena poziomu bólu wg scali VAS.
3. Ocena poziomu wypalenia zawodowego (ABQ – Athlete Burnout Questionnaire).

**Program usprawniania:**

Elementy indywidualnego programu postępowania fizjoterapeutycznego:

1. Kinezyterapia,
2. Elementy metod fizjoterapeutycznych,
3. Aplikacje dynamicznego plastrowania.

W czasie eksperymentu będą wykonywane aplikacje u badanych z dolegliwościami bólowymi. Dla każdego pacjenta aplikacje będą wykonywane trzykrotnie w odstępach siedmiodniowych.

Badania będą wykonywane przed terapią u osób z dysfunkcją narządu ruchu i po trzech seriach aplikacji metody dynamicznego plastrowania.

Program postępowania usprawniającego będzie realizowany osobiście przez wnioskodawcę. Będzie on dobierany indywidualnie do każdego pacjenta w zależności od: lokalizacji dolegliwości bólowych oraz zaburzeń motorycznych stwierdzonych podczas badania wstępnego u tancerzy.

1. Badani uczestniczący w eksperymencie będą ubezpieczeni na zasadach ogólnych w ramach zbiorowej umowy OC.
2. Wszyscy badani przed rozpoczęciem eksperymentu będą poinformowani o przeprowadzanym badaniu i związanych z tym ich prawach oraz będą zobowiązani do podpisania „Formularzu świadomej zgody pacjenta”.
3. Spodziewane korzyści dla pacjentów:
   - dodatkowe badania i testy funcjonalne,
   - kompleksowe badanie w kierunku hipermobilności,
   - kompleksowa ocena postawy ciała,
   - normalizacja napięcia mięśni u pacjentów z nadruchomością stawów,
   - zmniejszenie bólu,
   - wydłużone oddziaływanie terapeutyczne do 24 godz. na dobę;
4. Zagrożenia wynikające dla pacjentów z przeprowadzonego eksperymentu: nie zakłada się.
5. Uzyskane wyniki badań zostaną poddane analizie statystycznej.

……………………………………………….

Kierownik Kliniki/Zakładu,

w której prowadzone będzie badanie.

**Wzór świadomej zgody pacjenta na udział w badaniu**

**ZGODA na udział w badaniu naukowym:**

**Wpływ aplikacji metody dynamicznego plastrowania na dysfunkcje narządu ruchu u tancerzy.**

............................................

(Imię i nazwisko badanego)

.................................................................................................................................................

(Adres zamieszkania)

.......................................................

(pesel badanego)

**Oświadczenie**

Niniejszym oświadczam, że wyrażam zgodę na dobrowolne poddanie się badaniu medycznemu. Wiem, że moje dane personalne nie zostaną ujawnione, a zebrane informacje pozostaną poufne. Wyrażam zgodę na ujawnienie zebranych informacji medycznych osobom uczestniczącym w prowadzeniu badania. Stwierdzam, że nie będę sprzeciwiać się w wykorzystywaniu wyników badania.

Jednocześnie oświadczam, że zapoznałam się z „Informacją dla pacjenta”. Miałam/em sposobność zadawania pytań i uzyskałam/em zadawalające odpowiedzi. Mój podpis na niniejszym formularzu został złożony dobrowolnie. Zostałam/em poinformowana, że mogę odmówić zgody na udział w badaniach lub cofnąć ją w każdej chwili, a także podczas wykonywania badań, bez jakichkolwiek konsekwencji, czy zmiany sposobu leczenia. Wyrażam zgodę na przetwarzanie danych osobowych w związku z prowadzonym programem badawczym.

„Przeczytałam/em i akceptuję”

............................................

(data)

............................................ .......................................................

(podpis badanego) (podpis prowadzącego badanie)

**INFORMACJA DLA OSOBY BADANEJ**

Eksperyment badawczy prowadzony przez fizjoterapeutę mgr Marlenę Drężewską w Kieleckim Teatrze Tańca dotyczy tancerzy i uczniów tańca.

W skład badań wchodzi wywiad personalny, czyli podstawowe informacje o osobie badanej: dane osobowe, wiek, obecność dolegliwości bólowych, przebyte choroby/urazy oraz badanie przedmiotowe polegające na ocenie postawy ciała, badaniu ruchomości oraz badaniu sprawności kończyn dolnych, miednicy i dolnego odcinka kręgosłupa (pomiar zakresów ruchu i siły mięśniowej).

Badani z dolegliwościami bólowymi będą poddani terapii Kinesiotapingu polegająca na stosowaniu aplikacji taśmą Kinesio Tex oraz kinezyterapii. Celem terapii jest redukcja bólu oraz zmniejszenie napięcia mięśniowego.

Spodziewane korzyści dla pacjentów: dodatkowe badania i testy funcjonalne, kompleksowa ocena postawy ciała, indywidualnie dobrany program usprawnienia, normalizacja napięcia mięśni u pacjentów z nadruchomością stawów, zmniejszenie bólu, zastosowanie nowej, bezinwazyjnej metody terapeutycznej, poprawa jakości życia.

Zagrożenia wynikające dla pacjentów z przeprowadzonego eksperymentu: nie zakłada się.

Marlena Drężewska Kielce 01.12.2012r.

ul. Piastowska 20B

07-407 Czerwin

Wyrażam zgodę na przetwarzanie moich danych osobowych dla celów pracy doktorskiej.
