# Supplementary material for: Hypermobility of joints in dancers
Source: PLoS One. 2019 Feb 22;14(2):e0212188. doi: 10.1371/journal.pone.0212188 (PMC6386248; doi:10.1371/journal.pone.0212188)
Supplement: S2 File — (DOCX) [file pone.0212188.s003.docx]

Marlena Drężewska Appendix 1

ul. Piastowska 20B (To be completed by the Bioethical Committee)

07-407 Czerwin Date of proposal_________________

No. of the act_________________________

**PROPOSAL
To the Bioethical Committee on expressing opinions on conducting
evaluation of a scientific study.**

1. **The research is directed by Zbigniew Śliwiński, PhD.**

The experiment will be carried out at the Kielce Dance Theater, Plac Moniuszki 2B
25-334 Kielce.

The place of carrying out the trial is dictated mainly by the possibility of obtaining a research group to carry out a research experiment. The applicant will perform the test herself.

1. **Topic manager (applicant) and members of the research team:**

Applicant: Marlena Drężewska - MA physiotherapy.

Team:
Zbigniew Śliwiński, PhD

Wojciech Kiebzak, PhD

Małgorzata Starczyńska, PhD

Marek Woszczak, PhD

Marek Kiljański, PhD.

1. **Project title:**

Impact of the application of the Kinesiology Taping on the dysfunctions of the motor system in dancers.

1. **Proposed date of completion of trial:**

2013r.

1. **Kinesiology Taping**

The creator and propagator of the revolutionary method of treatment called Kinesiology Tapingiem is Kenzo Kase - Japanese chiropractic, President of the Kinesio Taping Society and the National School of Chiropractic in Japan, a graduate of the University of Meiji and the Chicago Chiropractic National School.

The assumption of the method was that the applied therapy could have a detrimental effect on the patient not only during the visit, but also after its completion, because the positive physiotherapeutic effects disappear frequently with the passage of time.

The idea of ​​creating an acceptable body support and therapy required appropriate materials. Initial attempts to use non-stretchable tapes in athletes and patients did not give the desired results. It has been developed, in the course of many years of experience, a patch with the name Kinesiology Tape, whose thickness, specific gravity and extensibility - in the range of 130-140%, are similar to those of human skin. It is waterproof, permeable to air, which allows for uninterrupted heat exchange. The basis for kinesiotaping is completely different from the effect of sports taping.

Kinesiology Taping is primarily a sensory interaction. Applications allow to maintain full range of motion, normalize muscle tension, activate damaged muscles, reduce pain, and also eliminate stasis and lymphatic edema, correct fascia and skin alignment, and improve microcirculation.

Each application is conditioned by clinical indications. Plaster Kinesiology Tape glued in the right way most often in a position in which the skin and fascia is tense creates a surface corrugation, increasing the space between the skin and fascia, which improves blood and lymph microcirculation and activates pro-self healing. The disorder of facial fascia coexisting with many disorders is a mechanism that hinders self-healing. Therefore, the impact on the reduction of pressure will facilitate the subcutaneous flow and the expansive displacement of tissues.
The following techniques are used in the Kinesiology Taping method: muscular, ligamental, fascial, lymphatic, functional and corrective.

Kinesiology Taping affects the function of the muscles, improving their functioning, especially when they are excessively stretched, reducing pain and increased tension, reducing fatigue and increasing the range of motion in the joints affected.

Thanks to this method, physiotherapy has gained a new effective therapeutic tool. Observing the possibilities that the method brings, we find application for areas not yet available for physiotherapy.

**Joint Hypermobility Syndrom (JHS)**

Joint Hypermobility Syndrome (JHS) is a generalized congenital failure of the connective tissue of the whole organism, associated with the disturbances in the proportion of collagen type I and III, causing symptoms in the organ of movement and a number of abnormalities associated with other systems containing connective tissue.

The main clinical symptoms in the musculoskeletal system (constitutional hypermobility of the joints) are the laxity of ligaments and joint capsules of the peripheral and intercostal joints of the spine and the range of joint movements increased in relation to the norm, resulting in lowering the mechanical and stabilizing capacity of the intervertebral discs and joints.

In the clinical diagnosis of JHS, different scales are used, among others Sachse, Beinghton, Hakim and Graham.

Increased mobility in the joints can be a congenital change, but also acquired - in the course of many years of intense training, as a result of an injury, as well as other diseases of connective tissue and / or motion system. The biggest problem is the diagnosis of the team of JHS in people in whom an increased range of movements in the joints is the effect of many years of training, eg in dancers or gymnasts.

**Research hypotheses:**

Due to the frequent occurrence of Joint Hypermobility Syndrom in dancers and various types of dysfunctions of the musculoskeletal system connected with the occurrence of pain - often preventing the participation of dancers in training or performances, it was decided to verify the following research hypotheses:

1. Most dancers in different age groups have observed constitutional or acquired hypermobility
2. Adaptation of appropriate criteria for the assessment of JHS is necessary to create an individual program to improve dancers.

3. Applications of Kinesiology Taping normalize muscle tone in dancers.

4. Kinesiology Taping reduces the level of pain experienced by dancers.

5. Techniques of the Kinesiology Taping method improve the stability of peripheral peripheral joints in dancers.

6. Movement dysfunctions affect the mood level of dancers.

**Material of the trial:**

A group of about 80 dancers of both sexes will be subjected to the study. The subjects will be dancers of the Kielce Dance Theater.

**Methods:**

The study will include:
1. Personal interview.
2. Assessment of the body posture of dancers with particular emphasis on pelvis statics.
3. Execution of 13 Sachse tests defining either non-constitutional hypermobility, including three assessment categories:
• category A - mobility of joints ranges from hypomobility to normal mobility.
• category B - normal mobility or low hypermobility.
• category C - significantly increased mobility, hypermobility.
4. The Beighton scale.
5. The Hakim and Graham tests.
6. Performing screening tests of the dynamic slicing method for the lower body area:
• Linder 2 test,
• Abdominal compression test
• Patric Fabre test
• SLR test.

1. Measuring muscle strength using the MICROFET 2 - Hoggan Health Industries, Inc.
   During the test, the subject will in an isolated position. The researcher with one hand stabilizes the patient's position and with the other hand he holds the device's transducer on the examined muscle. The subject performs the tension of the indicated muscle and a typical movement for this muscle.

The test will concern the following muscles:

m. najszerszy grzbietu

- m. quadratus lumborum,

- m. piriformis,

- m. gluteus medius,

- m. gluteus maximus,

- m. iliopsoas,

- m. rectus femoris,

- m. biceps femoris,

- m. semimembranosus,

- m. semitendinosus,

- m. tensor fascie latae,

- m. adductor brevis,

- m. adductor longus,

- m. adductor magnus.

8. Measurement of the range of movements (ROM) of the lower limb and the lumbar region of the spine using a digital inclinometer. The range of movements will be determined using the ISOM (International Standard Orthopedic Measurement) method, and the results will be recorded using the SFTR system (Sagital, Frontal, Transverse, Rotation).

9. Assessment of pain level according to the VAS scale.

10. Assessment of the level of professional burnout (Athlete Burnout Questionnaire – ABQ).

**Improvement program:**

Elements of an individual physiotherapeutic program:
a) Kinesitherapy,
b) Elements of physiotherapeutic methods,
c) Kinesiology Taping applications.

During the experiment, applications will be made to subjects with pain. For each patient, applications will be performed three times in seven-day intervals. The tests will be performed before therapy in patients with motor dysfunction and after three series of applications of the method of Kinesiology Taping.

The improvement procedure program will be implemented personally by the applicant. It will be selected individually for each patient depending on: the location of pain and motor disorders found during the preliminary examination in dancers.

11. The participants will be insured on general terms under a collective civil liability contract.

12. All subjects before the start of the experiment will be informed about the study and related rights, and will be required to sign the "Informed consent form".

13. Expected benefits for patients:

- additional tests and functional tests,

- comprehensive research towards hypermobility,

- comprehensive assessment of body posture,

- normalization of muscle tone in patients with articular hyperplasia,

- reduction of pain,

- extended therapeutic effect up to 24 hours per day.

14. Threats resulting from patients in the experiment: it is not assumed.

15. The obtained test results will be subjected to statistical analysis.

.......................................................
Head of the Clinic / Department, where the test will be conducted.
